# Optimization of Purification, Identification and Evaluation of the *in* *Vitro* Antitumor Activity of Polyphenols from *Pinus Koraiensis* Pinecones

**DOI:** 10.3390/molecules200610450

**Published:** 2015-06-05

**Authors:** Juanjuan Yi, Zhenyu Wang, Haina Bai, Xiaojin Yu, Jing Jing, Lili Zuo

**Affiliations:** 1 Harbin Institute of Technology, 73 Huanghe Road, Nangang District, Harbin 150090, China; 2 Northeast Forestry University, 26 Hexing Road, Xiangfang District, Harbin 150040, China; 3 Heilongjiang University of Chinese Medicine, 24 Heping Road, Xiangfang District, Harbin 150040, China; 4 Jilin Medical College, 5 Jilin street, Jilin 132013, China

**Keywords:** pinecone of *Pinuskoraiensis*, polyphenols purification, response surface methodology, antiproliferative activity, compound identification

## Abstract

In this study, an efficient purification method for the polyphenols of *Pinus koraiensis* pinecone (PPP) has been developed. AB-8 resin was verified to offer good adsorption and desorption ratio for PPP. Response surface methodology (RSM) indicated that the optimized purification parameters for PPP were 1.70 mg GAE/mL phenolic sample concentration, 22.00 mL sample volume, and 63.00% ethanol concentration. Under these conditions, the experimental purity of PPP was 27.93 ± 0.14% (*n* = 3), which matched well with the predicted purity of 28.17%. Next, the antiproliferative effects of PPP on seven cancer cell lines, including A375 (human skin melanoma cancer cell line), A549 (human lung cancer cell line), SH-SY5Y (human neuroblastoma cell line), LOVO (human colon cancer stem cell line), MCF-7 (human breast cancer cell line), HeLa (human cervical cancer line), and HT29 (human colon cancer line), were examined by MTT assays. The results indicated that PPP had the highest capacity for inhibiting LOVO cells growth with an EC_50_ value of 0.317 ± 0.0476 mg/mL. Finally, Ultra-high performance liquid chromatography- tandem mass spectrometry (UPLC-MS) was used to tentatively identify twenty-four peaks in the purified PPP, of which five representative peaks were identified as catechin, methyl quercetin, *o*-vanillin, luteolin and coronaric acid. Our results demonstrate that *Pinus koraiensis* pinecone is a readily available source of polyphenols, and the purified PPP could be a promising natural antitumor agent for applications in functional foods.

## 1. Introduction

Cancer, with millions of new cancer patients diagnosed each year, remains a leading cause of mortality, second only to heart diseases [1,2]. A recent report reveals that tumor promotion is the only reversible event during cancer development [3]. Therefore, early intervention targets will inhibit cancerous cell proliferation. Moreover, epidemiology suggests dietary consumption of plant polyphenols is associated with a lower incidence of cancer [4]. Therefore, great efforts have been made to develop new antitumor agents from natural products [5,6].

*Pinaceae* *koraiensis* is a kind of evergreen tree which belongs to the *Pinus* genus of the family *Pinaceae*. It is native to eastern Asia, and widely distributed in the northeast China [7]. Pine polyphenols, secondary plant metabolites, are important determinants of the nutritional qualities of *P*. *koraiensis* [8]. Pine polyphenols have been attracting the attention of many biologists due to their biological activities [9], such as anti-oxidant [10], anti-tumor [11], anti-microbial properties [12] and so on. In addition, *P. koraiensis* pinecone, a by-product in the processing of *P. koraiensis* seeds, has been studied partly due to its potential as a rich source of polyphenol compounds. However, researches on *P. koraiensis* pinecone have mainly focused on the separation of its biologically active compounds [13,14] as crude polyphenols always contain chlorophyll, proteins, polysaccharides and other impurities, which limit their applications, therefore it is urgent to develop an efficient purification method to obtain a high purity form of PPP, so as to study its antitumor effects.

The purification methods for polyphenols mainly include liquid-liquid extraction [15], supercritical fluid extraction [16,17], membrane filtration [18,19,20], ion exchange [21] and resin adsorption [22,23,24,25]. Comparatively, the resin adsorption method is the most suitable one for the separation and purification of biologically active substances due to its low cost, high efficiency and simple procedure [26,27]. In particular, AB-8 macroporous resin has been widely used in purification of polyphenols because of its appropriate surface area and nuclear pore size [23,28]. However, there is only limited information on the purification of PPP by AB-8 macroporous resin.

Response surface methodology (RSM) is an effective statistical technique for optimizing complex processes. It can reduce the number of experimental trials needed to evaluate multiple parameters and their interactions [29]. Moreover, Box-Behnken design (BBD), as a type of response surface design, is also widely used in many researches [30,31,32,33]. Therefore, the first aim of the present study was to optimize the purification parameters of the polyphenol extracts of *P. koraiensis* pinecone (PPP) by RSM and investigate the antiproliferative properties of PPP on seven cancer cell lines. Next, HPLC-MS analyses were performed to identify the components of purified PPP. The overall aim of this study was to supply some new information on the purification and antiproliferative activity of PPP for the development and application of *P. koraiensis* pinecones.

## 2. Results and Discussion

### 2.1. Adsorption and Desorption on AB-8 Resin

**Figure 1:**
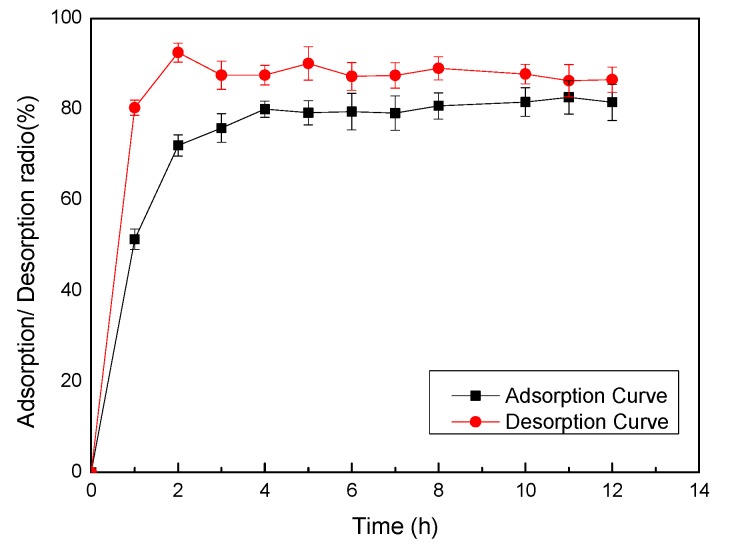
Static adsorption and desorption ratios of AB-8 resin at different time. Results were shown as means ± standard deviation (*n* = 3).

The adsorption and desorption performance relates to the capabilities of the macroporous resins and chemical features of the absorbed substance [34]. The adsorption and desorption ratios of PPP on AB-8 resin were verified and shown in Figure 1.

Results indicated that AB-8 resin possessed a high adsorption ratio and a higher desorption ratio, therefore, AB-8 resin was verified to be suitable for purification of PPP. More specifically, the adsorption ratio of PPP increased sharply during the first 2 h, and then continued to increase much more slowly. After 4 h, the adsorption level reached equilibrium which was likely due to the moderate polarity of AB-8 resin [35]. Therefore, the adsorption equilibrium was set at 4 h for later studies. Meanwhile, the desorption ratio also increased rapidly within 2 h to reach a maximum value and stay stable.

### 2.2. Effect of Sample Phenolic Concentration on Purity of PPP

The purification process was carried out using a range of sample phenolic concentrations from 1.0 to 3.5 mg GAE/mL with a sample volume and an ethanol concentration of 30 mL and 60% (*v*/*v*), respectively. The effect of sample phenolic concentration on the purity of PPP is shown in Figure S1A (Supplementary Material). It was found that phenolic purity increased quickly to a maximum value of 25.08% ± 0.81% as sample phenolic concentration increased from 1.0 to 1.5 mg GAE/mL, and then dropped rapidly until sample phenolic concentration exceeded 2.0 mg GAE/mL before descending slowly. The results indicated that sample phenolic concentration of 1.5 mg GAE/mL was optimal to obtain PPP of high purity.

### 2.3. Effect of Sample Volume on Purity of PPP

In this study, sample volumes were set at 10, 20, 30, 40, 50, and 60 mL while other parameters were set as follows: sample phenolic concentration of 1.5 mg GAE/mL and ethanol concentration of 60%. As shown in Figure S1B (Supplementary Material), the purity significance was almost the same with a sample volume of 10 or 20 mL, and the maximum purity of PPP was observed between 10 and 20 mL. The highest purify was obtained with the optimized sample volumes, when the experimental error was taken into consideration. If the sample volume is too small, the error of the result would likely be bigger, so 20 mL was used in the experiments.

### 2.4. Effect of Ethanol Concentration on Purity of PPP

Purification processes were carried out at different ethanol concentrations of 20%, 30%, 40%, 50%, 60% and 70%, while sample phenolic concentration and sample volume were fixed at 1.5 mg GAE/mL and 20 mL, respectively. The effect of ethanol concentration on purity of PPP is shown in Figure S1C (Supplementary Material). The variance of phenolic purity increased first and then decreased with the increase of ethanol concentration, and peaked at 60%. The results indicated that 60% ethanol was the most suitable for the purification of PPP, which may be related to the solvent polarity and the solubility of PPP.

### 2.5. Optimization of Purification Parameters for PPP

#### 2.5.1. Statistical Analysis and the Model Fitting

A total of 15 runs were performed to optimize the three individual parameters in the current BBD as shown in Table 1.

**Table 1:** Box-Behnken experimental design with the independent variables (*n* = 3).

Run	X_1_ (mg/mL)	X_2_ (mL)	X_3_ (%)	Phenolic Purity (%)
1	0	0	0	27.45
2	−1	0	−1	15.46
3	0	−1	1	17.62
4	1	0	−1	19.21
5	−1	−1	0	13.59
6	−1	0	1	17.11
7	0	−1	−1	15.60
8	1	0	1	24.90
9	1	1	0	24.41
10	0	0	0	27.14
11	1	−1	0	16.61
12	−1	1	0	16.35
13	0	1	1	23.36
14	0	1	−1	18.16
15	0	0	0	26.48

X_1_: sample phenolic concentration; X_2_: sample volume; X_3_: ethanol concentration.

The model for the response variable could be expressed by the following quadratic polynomial equation in the form of coded values:

Y = 27.02 + 2.83X_1_ + 2.36X_2_ + 1.82X_3_ + 1.26X_1_X_2_ + 1.01X_2_X_3_ + 0.80X_1_X_3_ − 4.40X_1_^2^ − 4.89X_2_^2^ − 3.45X_3_^2^

where Y is the purity of PPP, and X_1_, X_2_ and X_3_ are the coded variables for sample phenolic concentration, sample volume and ethanol concentration, respectively.

Analysis of variance (ANOVA) for the fitted quadratic polynomial model of purification of PPP is shown in Table 2. The determination coefficient R^2^ was 0.9965 and suggested that 99.65% of the variations could be illustrated by the fitted model [36]. As shown in Table 2, the value of R^2^_adj_ was 0.9901 and was close to R^2^, which revealed that the model was highly significant. At the same time, the coefficient of variation (CV = 2.38%) was a relatively small value which indicated a better reliability of the experimental values. Significance of model was also determined by lack-of-fit test. Table 2 shows *F*-value and *p*-value of the lack of fit were 0.91 and 0.5629, respectively, which suggested that the lack of fit was not significant and the model was satisfactory. Moreover, the significance of each coefficient was also checked by the *p*-value. Table S2 (Supplementary Material) showed the cross product coefficients (X_2_X_3_) were significant with small *p*-values (*p* < 0.05). The other term coefficients were also very significant (*p* < 0.01). These results suggested the model could be used to predict these responses.

**Table 2:** Analysis of variance for the fitted quadratic polynomial model of purification of PPP.

Source	SS ^a^	DF ^b^	MS ^c^	*F*-Value	Prob-*F*
Modle	325.17	9	36.13	156.06	<0.0001
Residual	1.16	5	0.23		
Lack of fit	0.67	3	0.22	0.91	0.5629
Pure error	0.49	2	0.25		
Cor.total	326.33	14			
R^2^ = 0.9965; R^2^_adj_ = 0.9901; CV = 2.38					

^a^ Sums of squeares, ^b^ Degree freedom, ^c^ Mean square.

#### 2.5.2. Optimization of the Purification Conditions

**Figure 2:**
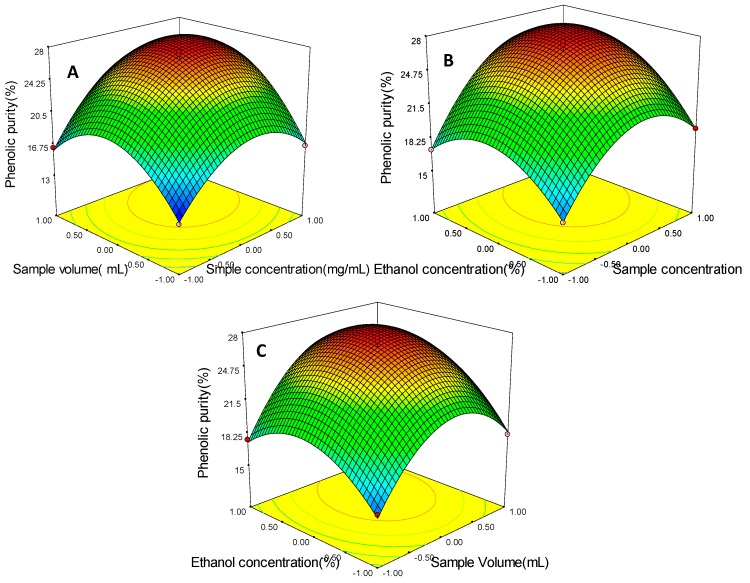
Response surface plots showing the effect of sample phenolic concentration (mg/mL), sample volume (mL) and ethanol concentration (%) on the phenolic purity (%). (**A**) the effect of sample phenolic concentration (X1) and sample volume (X2); (**B**) the effect of sample phenolic concentration (X1) and ethanol concentration (X3); (**C**) the effect of sample volume (X2) and ethanol concentration (X3).

The 3D surface plots, which provide a method to visualize the relationship between responses and experimental levels of each variable [29] are shown in Figure 2. Figure 2A gives the ethanol concentration X_3_ (0 level) and shows the effects of sample phenolic concentration X_1_ and sample volume X_2_ on the phenolic purity. As the sample volume increased, the phenolic purity increased quickly at first until reaching a maximum and then decreased when the sample phenolic concentration was at a low level. Similarly, the increase of sample phenolic concentration caused an initial increase and a later decrease in the phenolic purity. This result indicated the phenolic purity was affected significantly by sample phenolic concentration and sample volume, which was also confirmed by the results in Table S2 (Supplementary Material).

Figure 2B shows a great increase in phenolic purity with an increase in sample phenolic concentration from 1.00 to 1.66 mg GAE/mL, and then a gradual decrease. The phenolic purity slowly increased then decreased with the increasing of ethanol concentration. These results indicated that 1.66 mg GAE/mL and 63.14% were required to achieve maximum increase.

Figure 2C shows that the phenolic purity increased steadily with increasing ethanol concentration from 50% to 63.14%, then decreased slowly. Similarly, phenolic purity increased rapidly with sample volume from 10 mL to 21.65 mL and decreased rapidly after 21.65 mL, which indicated 21.65 mL was required to obtain maximum increase. According to Figure 2 above, it can be concluded that optimal purification conditions of PPP were sample phenolic concentration of 1.66 mg GAE/mL, sample volume of 21.65 mL and ethanol concentration of 63.14%. Among the three purification parameters studied, sample phenolic concentration was the most significant factor affecting the phenolic purity according to the gradient of the slope in the 3D response surface plot.

### 2.6. Verification of Predictive Model

A high purity PPP was obtained with the optimized purification parameters, where the feasibility of the experiment was taken into consideration, so the suitability of the model equation for predicting the optimum response values was tested by using the optimal conditions with small modifications. Table 3 shows the maximum predicted and experimental phenolic purity. By using the predicted optimum conditions, the model predicted accordingly a maximum response of 28.17%. In order to ensure the predicted result was not biased toward the actual value, experiment rechecking was performed by using these modified conditions: sample phenolic concentration of 1.70 mg GAE/mL, sample volume of 22.00 mL and ethanol concentration of 63.00%. The mean value of 27.93 ± 0.14 (%) (*n* = 3) obtained from real experiments, demonstrated the validation of the RSM model. The analysis results indicated that the model of Equation (4) was satisfactory and accurate.

**Table 3:** Predicted and experimental values of the responses at optimum and modified conditions. The actual experiment results were the means ± standard deviation of three independent experiments (*n* = 3).

	Sample Phenolic Concentration (mg/mL)	Sample Volume (mL)	Ethanol Concentration (%)	Phenolic Purity (%)
Optimum Conditions (predicted)	1.66	21.65	63.14	28.17
Modified Conditions (actual)	1.70	22.000	63.00	27.93 ± 0.14

### 2.7. Antiproliferative Activity of PPP

**Figure 3:**
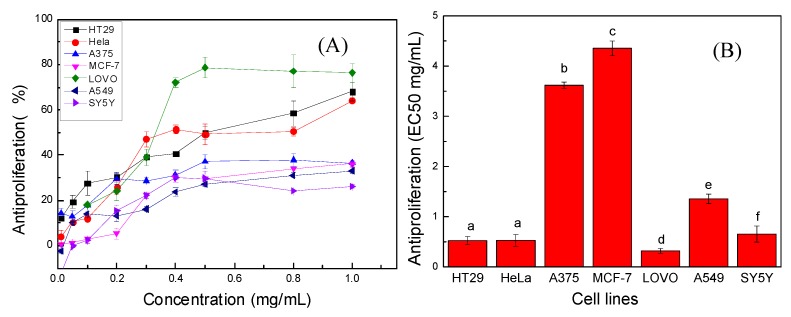
Antiproliferative effects of PPP on seven cancer cells. (**A**) Inhibition of seven cells proliferation with treatment of different concentrations of PPP; (**B**) The EC_50_ values for the inhibition of seven cells growth by PPP. Results were represented as means ± standard deviation of three parallel measurements followed by the different letters, which indicated significantly different (*p* < 0.05), (*n* = 3).

Next, the antiproliferative activities of PPP were evaluated against the A375, A549, SH-SY5Y, LOVO, MCF-7, HeLa and HT29 cell lines by MTT assays. There was a considerable difference in the sensitivity of the seven cancer cell lines towards PPP, as presented in Figure 3A. Overall, PPP roughly inhibited the proliferation of the seven kinds of cells in dose dependent manner. It showed strong anti-proliferative effects on the three cancer cell lines of LOVO, HT29 and HeLa with antiproliferatition rates of 80%, 70% and 65%, respectively, when the PPP concentration was 1.0 mg/mL. For the rest of the cell lines (A375, MCF-7, A549 and SY5Y), PPP showed relatively poor inhibition effects with no higher than 40% anti-proliferation rates. Phenolic-rich extracts have been widely investigated for their potential antiproliferative properties against a variety of cancer cells, and some of them also have shown promising results [37,38,39].

Antiproliferative activity was expressed as the 50% effective concentration (EC_50_), where a low EC_50_ value means a high antiproliferative value. Based on the EC_50_ values in Figure 3B, the antiproliferation effects of the PPP on the seven cell lines were in the following decreasing order: MCF-7 > A375 > A549 > SH-SY5Y > HeLa > HT29 > LOVO. The antiproliferative ratio of PPP against LOVO cells was the highest (Figure 3A), and the EC_50_ value of the LOVO cell line (0.317 ± 0.0476 mg/mL) was also the lowest accordingly in all the cancer cell lines (*p* < 0.05) (Figure 3B).

In our study, PPP showed different antiproliferative effects on the seven target cancer cell lines, which indicated that different cancer cells had different sensibility against the same test sample. This is in accordance with report of Fan *et al* that human liver cancer HepG2 cells were more sensitive to fruit extract treatment than colon cancer HT-29 cells [40]. Furthermore, the PPP concentrations employed in the assay have been normalized, suggesting that the different antiproliferative responses on seven cells were associated with the polyphenol compositions of PPP, rather than the polyphenol contents. Thus LOVO cells were inferred to be more vulnerable to the major polyphenols from *Pinus koraiensis* in our study.

### 2.8. UPLC-Q-TOF-MS Analysis of Purified PPP

**Figure 4:**
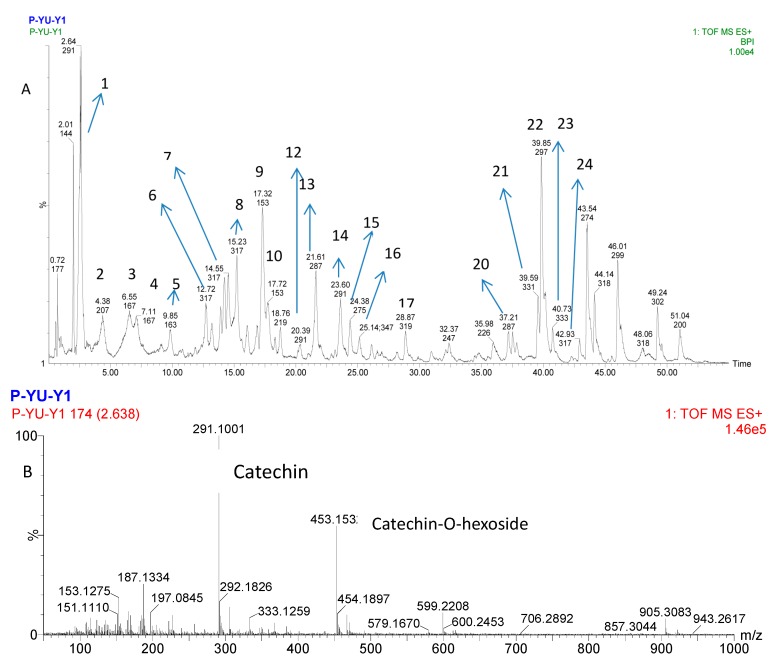

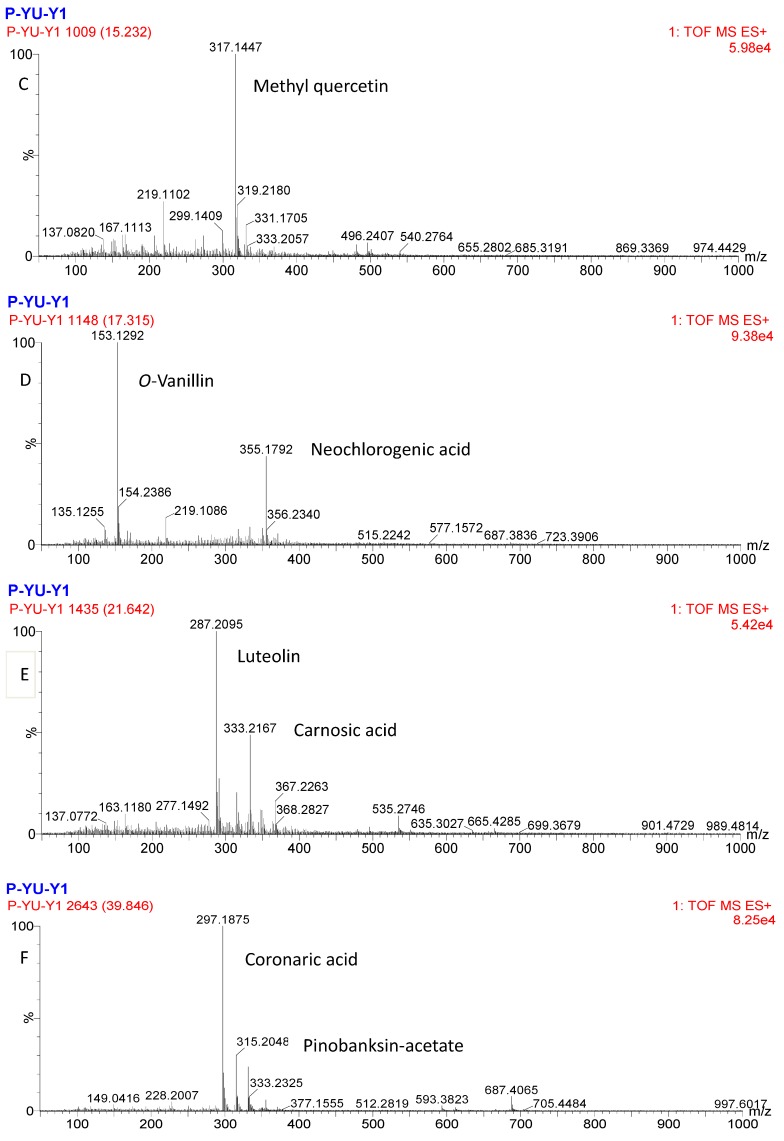
UPLC-Q-TOF-MS total ion chromatogram (TIC) of purified PPP and the ion chromatograms of representative peaks. (**A**) Total ion chromatographic profiling of purified PPP; (**B**) MS/MS spectrum of peak 1; (**C**) MS/MS spectrum of peak 8; (**D**) MS/MS spectrum of peak 9; (**E**) MS/MS spectrum of peak 13; (**F**) MS/MS spectrum of peak 22. Mass spectral data positive mode of phenolics.

Tentative characterization of purified PPP was generated based on elemental composition data determined by UPLC-MS. Figure 4A shows the total ion chromatogram (TIC) of purified PPP.

The phenolic compounds were tentatively identified on the basis of comparisons of their retention time and mass spectra with literature data [41,42,43,44,45,46,47,48,49,50,51]. The TIC obtained revealed the presence of twenty-four peaks in the purified PPP and listed in Table 4. Five representative peaks identified as catechin, methyl quercetin, *o*-vanillin, luteolin and coronaric acid, respectively, are in Figure 4B–F.

As reported in the literature [41,42,43], the UPLC catechin elutes prior to epicatechin, thus, peak 1 and 12 (14) were identified as catechin (Figure 4B) and epicatechin, respectively. Peaks 6, 7, 8 and 24 with [M + H]^+^ at *m/z* 317 were identified as 3-hydroxy-4-*O*-β-glucosylbenzoic acid, isorhamnetin, methyl quercetin (Figure 4C) and tamarixetin according to previous reports and the obtained mass spectra [42,43,44,45]. Both peaks 9 and 10 yielded *m/z* 153 [M + H]^+^ peaks, and *o*-vanillin (Figure 4D) and *p*-vanillin were identified by examining the known literature data [46]. Peaks 13 (*m/z* 287) and 22 (*m/z* 297) were characterized as luteolin and coronaric acid, respectively (Figure 4E–F) [45,47,48]. In addition, by comparing the mass spectra with literatures, peaks 16 and 17 were also identified as isorhamnetin, rosmanol isomer and myricetin [46,49]. Peaks 20, 21 and 23 showed [M + H]^+^ peaks at *m/z* 287, 331 and 333, which was identified as kaempferol, carnosol isomer and monogalloyl glucose, respectively [44,49]. Identifications of other peaks were strongly correlated with the studied references values [41,42,45,47,50,51].

**Table 4:** Identification of purified PPP by UPLC-MS. Mass spectral data positive mode.

Peak	t_R_ (min)	MS (*m/z)*	MW	Molecular Formula	Identification
1	2.64	291	290	C_15_H_14_O_6_	Catechin
2	4.38	207	206	C_11_H_10_O_4_	Scopoletin + CH2
3	6.55	167	166	C_8_H_7_O_4_	Vanillic acid-H
4	7.11	167	166	C_8_H_7_O_4_	Vanillic acid-H
6	12.72	317	316	C_16_H_12_O_7_	3-Hydroxy-4-*O*-β-glucosylbenzoic acid
7	14.55	317	316	C_16_H_12_O_7_	Isorhamnetin
8	15.23	317	316	C_16_H_12_O_7_	Methyl quercetin
9	17.32	153	152	C_8_H_8_O_3_	*o*-Vanillin
10	17.72	153	152	C_8_H_8_O_3_	*p*-Vanillin
12	20.39	291	290	C_15_H_14_O_6_	Epicatechin
13	21.61	287	286	C_15_H_10_O_6_	Luteolin
14	23.60	291	290	C_15_H_14_O_6_	Epicatechin
15	24.38	275	274	C_15_H_14_O_5_	Phloretin
16	25.14	347	346	C_20_H_26_O_5_	Rosmanol isomer
17	28.87	319	318	C_15_H_10_O_8_	Myricetin
20	37.21	287	286	C_15_H_10_O_6_	Kaempferol
21	39.59	331	330	C_20_H_26_O_4_	Carnosol isomer
22	39.85	297	296	C_18_H_32_O_3_	Coronaric acid
23	40.73	333	332	C_16_H_12_O_6_	Monogalloyl glucose
24	42.93	317	316	C_16_H_12_O_7_	Tamarixetin

These identified polyphenol compositions may play a key role in the biological activities of PPP, especially, the catechin and epicatechin components (peak 1 and peak 12) may be closely related with the antitumor activity of PPP. The phenolic compositions of Annurca apple peel also showed that higher amounts of procyanidins (catechin and epicatechin) possessed the most effective antitumor function [52]. Some authors also have proved the antitumor activities of polyphenols were associated with their composition. Corsi *et al.* proved in their studies that gallic acid showed a higher inhibition capacity for HeLa cells than cinnamic acid [53]. The aqueous extract of carob pod was composed mainly by gallic acid and gallotannins, and exerted a good antiproliferative effect on T1 cell lines [54]. Anthocyanin-rich black currant extract and pitaya peel polyphenols significantly inhibited the growth of HepG2 cells and B16F10 melanoma cancer cells, respectively [55,56].

Moreover, these differences in functional activities also may be attributed to their different active components. Serra and others found that among phenolic compounds catechin and procyanidin B1 were the major contributors to the antioxidant activity of apples, whereas procyanidins (B1 and B2), phloridzin and epicatechin played an important role against human cancer cell proliferation [57].

## 3. Experimental Section

### 3.1. Samples and Chemicals

The dried pinecones of *P. koraiensis* were provided by the Forestry Bureau (Yichun, China). AB-8 macroporous resin was obtained from Soledad Technology Ltd. (Beijing, China) and was pretreated according to Sun *et al*. [29]. Roswell Park Memorial Institute 1640 (RPMI-1640) medium and fetal bovine serum (FBS) were provided from Hyclone Chemical Co. (Hyclone, Logan, UT, USA). Other chemicals were commercially available from local suppliers.

### 3.2. Extraction Procedure of PPP

The dried pinecones of *P. koraiensis* were smashed and passed through 30 mesh sieves. The pinecone powders were added to 60% ethanol (1:20), and subjected to ultrasonic treatment at 50 °C and 400 W for 1 h, then the extraction above was repeated twice. The extracts were combined and separated by filtration on a Buchner funnel, then centrifuged at 4000 rpm for 10 min. The supernatant was concentrated by rotary evaporation to afford the crude PPP.

### 3.3. Determination of Total Phenolic Content (TPC)

TPC was determined by the Folin–Ciocalteu method using gallic acid as a standard. Briefly, 1.0 mL of sample solution was mixed with 1.0 mL of Folin-Ciocalteu reagent and reacted for 5 min at 25 °C. Then, 1.0 mL of saturated Na_2_CO_3_ (10% *w*/*v*) and 1.0 mL of distilled water were added. The reaction mixture was kept at 25 °C for 2 h in the dark. Absorbance was measured at 765 nm. TPC was expressed as mg gallic acid equivalent per mL of sample solution (mg GAE/mL).

### 3.4. Static Adsorption and Desorption of AB-8 Resin Test

The pretreated AB-8 resins (5.0 g) and 50 mL of PPP (1.5 mg GAE/mL) were added to a 250 mL air-tight flask. Then, the flask was shaken at 100 rpm for 12 h to reach the equilibrium. Next, the residual PPP solution was removed first. Then, the AB-8 resins were washed with distilled water and desorbed by 50 mL 60% (*v*/*v*) ethanol solution in a flask, which was shaken at 100 rpm for 12 h. The TPC of the solution was determined at 1 h intervals. The adsorption and desorption ratios were calculated using Equations (1) and (2), respectively:

(1) $$E=100\;(C_{0}-C_{t})/C_{0}$$

(2) $$D=100\;C_{d}V_{d}/[V_{0}(C_{0}-C_{e})]$$

where *E* is the adsorption ratio (%); *C*_0_ and *C_t_* are the TPC of the sample solution at beginning and at time *t*, respectively (mg GAE/mL); *D* is the desorption ratio (%), *C_d_* is the TPC of the desorption solution (mg GAE/mL); *V_d_* is the desorption solution volume (mL); *V*_0_ is the initial volume of PPP (mL); *C_e_* is the equilibrium TCP in the sample solution.

### 3.5. Determination of Total Phenolic Purity (TPP)

Briefly, the crude PPP was dynamically adsorbed on a glass column packed with AB-8 resin. Based on the result of the static AB-8 resin adsorption experiments, the equilibrium time was chosen as 4 h. Distilled water was first used to remove sugars, acids and other water-soluble compounds. Then, the crude PPP was eluted with ethanol at a constant flow velocity of 1.5 mL/min, and two bed volumes (BV) of phenolic eluent was collected and concentrated in a rotary evaporator to afford the purified PPP. TPP (%) was calculated as per Equation (3):

(3) $$Phenolic\;purity(\text{\%})=\left(\frac{Purified\;polyphenols\;weight}{total\;solids\;weight}\right)\times 100\text{\%}$$

### 3.6. Experimental Design

The single-test method was first used to determine the preliminary ranges of the purification variables. Then, BBD was employed to optimize three independent variables (sample phenolic concentration X_1_, sample volume X_2_, ethanol concentration X_3_) for the purification of PPP in this optimization study. Table S1 (Supplementary Material) shows the three levels of these independent variables. Phenolic purity (Y) was taken as a response for the design experiment shown in Table 1. The behavior of the system was explained by Equation (4):

(4) $$Y=A_{0}+\sum_{i=1}^{3}A_{i}X_{i}+\sum_{i=1}^{3}A_{ii}X_{i}^{2}+\sum_{i=1}^{2}\sum_{j=i+1}^{3}A_{ij}X_{i}X_{j}$$

where *Y* is the dependent variable, *A*_0_ is a constant, and A_i_, A*_ii_*, and *A_ij_* are coefficients estimated by the model. *X_i_* and *X_j_* are levels of the independent variables while *A*_0_, *A_i_*, *A_ii_*, and *A_ij_* are the regression coefficients for the intercept, linearity, square, and interaction, respectively.

### 3.7. Cell Culture

The A375, A549, SH-SY5Y cell lines were provided by Harbin Medical University. The MCF-7 cell line was obtained from School of Life Science of Harbin Institute of Technology. HeLa and T29 cell lines were preserved by the School of Food Science and Engineering of Harbin Institute of Technology. The seven cell lines were grown in RPMI-1640 containing 10% heat-inactivated FBS and maintained in a 5% CO_2_/37 °C incubator [58,59].

### 3.8. Measurement of Antiproliferation of PPP on Cancer Cells

The antiproliferative activities of samples were measured by the MTT assay. The cancer cell lines (1 × 10^5^ cells/mL) were transferred to 96-well flat-bottom plates containing RPMI-1640 medium. The cells were allowed to settle overnight. After incubation, the medium was changed to 100 μL of growth medium added with various concentrations of samples in each well and incubated for another 48 at 37 °C in 5% CO_2_. Then, 10 μL of MTT solution (0.5 mg/mL) was added to each well. After 4 h of incubation, the untrasformed MTT was carefully removed and the precipitated formazan was dissolved in the 150 μL of DMSO per well, then shaken for 15 min, Cell antiproliferation (%) was determined at 490 nm and was calculated by Equation (5) [40,60]. All samples were conducted in triplicate:

Antiproliferative rate (%) = 100 × (absorbance of control − absorbance of treated cells)/ absorbance of control
(5)

### 3.9. Identification of Purified PPP by UPLC-Q-TOF-MS

The purified PPP was analyzed using a UPLC (ACQUITY™, Waters Technologies, Milford, MA, USA) equipped with a HSS T3 column (2.1 × 100 mm, 1.8 μm, waters). Eluent (A) and eluent (B) were methanol and acidified water (0.1% formic acid, *v*/*v*), respectively. The elution program was as follows: 0–30 min (5%–25% A), 30–40 min (25%–35% A), 40–50 min (35%–50% A), 50–51 min (50%–5% A), 51–60 min (5% A). And the operating conditions were: flow rate, 0.16 mL/min; column temperature, 30 °C; injection volume, 10 μL. UV-Vis absorption spectra were monitored at 280 nm. The UPLC system was coupled to Xevo QTof MS (Waters Technologies) equipped with an ESI source. The analysis parameters were set using positive ion mode with spectra acquired over a mass rang from *m/z* 100 to 1200. The ESI-MS parameters were set to voltage, +4.5 kv; dring gas temperature, 325 °C; dring gas flow, 10L /min.

### 3.10. Statistical Analysis

Analysis of the experimental design and calculation of predicted data were carried out using Design Expert (version 7.0, Stat-Ease, Inc., Minneapolis, MN, USA) to estimate the responses to the independent variables. All analytical measurements were run at least in triplicate unless specified otherwise. Values were averaged and given with the standard deviation (± SD).

## 4. Conclusions

In this paper, AB-8 resin was first verified to possess good adsorption and desorption ratios for PPP purification. Then, RSM with BBD was used to optimize the experimental variables, and the optimal conditions determined were as follows: sample phenolic concentration of 1.70 mg GAE/mL, sample volume of 22.00 mL and ethanol concentration of 63.00%. Under these optimal conditions, a maximum phenolic purity of 27.93% ± 0.14% could be achieved. In addition, the *in vitro* antiproliferative activities of PPP on seven cancer cell lines were evaluated. The results showed that PPP had the strongest inhibition on the proliferation of LOVO in a dose-dependent manner (*p* < 0.05). This result also indicated that the same components of PPP possessed selective antiproliferative effects against different target cell lines. Finally, twenty-four peaks were tentatively identified by UPLC-MS, of which five representative peaks were identified as catechin, methyl quercetin, *o*-vanillin, luteolin and coronaric acid in the purified PPP. The results indicated that *Pinus koraiensis* pinecone is a valuable source of polyphenols. Furthermore, this study also provided some useful information for the development of PPP as an antitumor drug.

## Supplementary Materials

Supplementary materials can be accessed at: http://www.mdpi.com/1420-3049/20/06/10450/s1.

## Nonstandard Abbreviations

*Pinus koraiensis: P. koraiensis*; polyphenols of *P. koraiensis* pinecone: PPP; Response surface methodology: RSM; Box-Behnken design: BBD; Gallic acid equivalent: GAE; Bed volume: BV; High performance liquid chromatography-tandem mass spectrometry: HPLC-MS.
