# Supplementary material for: Optimization of Purification, Identification and Evaluation of the in Vitro Antitumor Activity of Polyphenols from Pinus Koraiensis Pinecones
Source: Molecules. 2015 Jun 5;20(6):10450–67. doi: 10.3390/molecules200610450 (PMC6272533; doi:10.3390/molecules200610450)
Supplement: Supplementary file 1 [file molecules-20-10450-s001.pdf]

Supplementary Material

Table S1. Independent variables their levels used for BBD.

Variables	Level		
	**−1**	**0**	**1**
Sample concentration X_1 (mg/mL)	1.0	1.5	2.0
Sample volume X_2 (mL)	10	20	30
Ethanol concentration X_3 (%)	50	60	70

Table S2. Regression coefficients of the predicted quadratic polynomial model.

Source	SS [a]	DF [b]	MS [c]	*F*-Value	Prob-Value
X_1	63.96	1	63.96	276.26	<0.0001
X_2	44.46	1	44.46	192.05	<0.0001
X_3	26.50	1	26.50	114.46	0.0001
X_1-X_2	6.35	1	6.35	27.43	0.0034
X_1-X_3	4.08	1	4.08	17.62	0.0085
X_2-X_3	2.53	1	2.53	10.92	0.0241
$X^2{}_1$	71.46	1	71.46	308.65	<0.0001
$X^2{}_2$	88.08	1	88.08	380.45	<0.0001
$X^2{}_3$	44.05	1	44.05	190.29	<0.0001

[a]: Sums of squeares; [b]: Sums of squeares; [c]: Mean square.

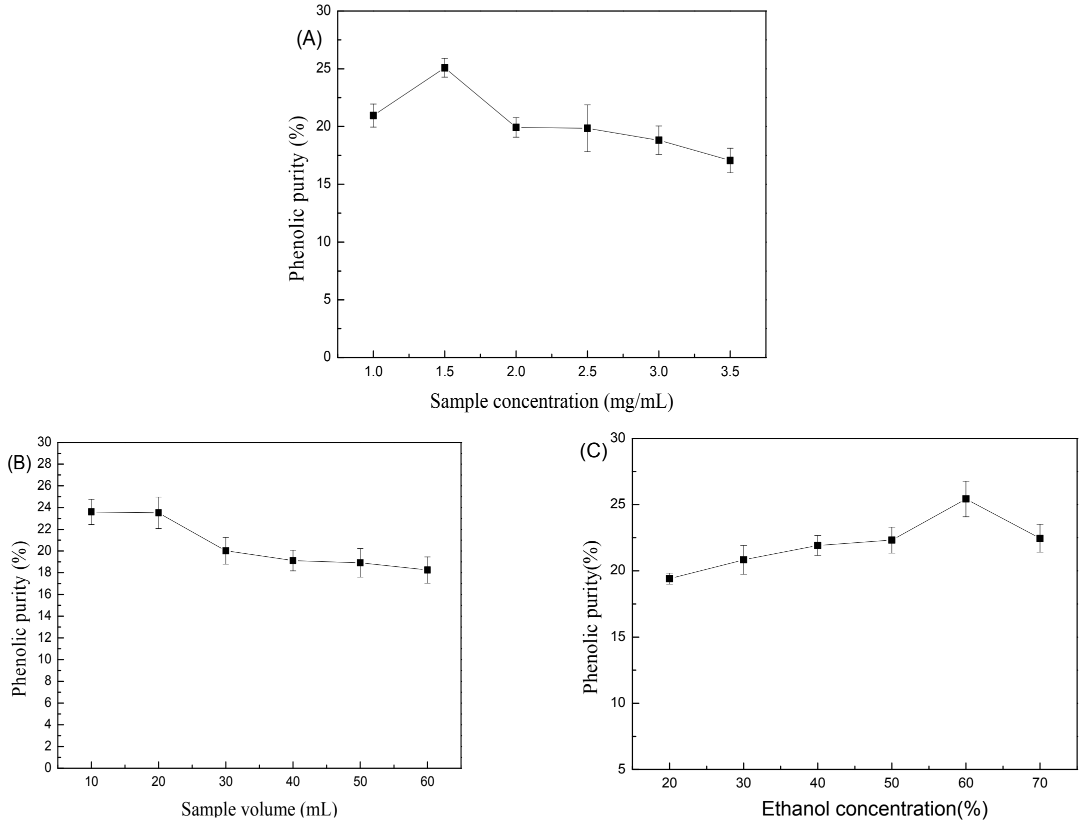

Figure S1. Effects of different purification parameters on purity of PPP (sample concentration, mg/mL; sample volume, mL; ethanol concentration, %).
